# New Trimethoprim-Like Molecules: Bacteriological Evaluation and Insights into Their Action

**DOI:** 10.3390/antibiotics10060709

**Published:** 2021-06-12

**Authors:** Marta Jorba, Marina Pedrola, Ouldouz Ghashghaei, Rocío Herráez, Lluis Campos-Vicens, Franciso Javier Luque, Rodolfo Lavilla, Miguel Viñas

**Affiliations:** 1 Laboratory of Molecular Microbiology & Antimicrobials, Department of Pathology & Experimental Therapeutics, Medical School, University of Barcelona, Bellvitge Institute for Biomedical Research (IDIBELL), Hospitalet de Llobregat, 08907 Barcelona, Spain; mjorba@ub.edu (M.J.); rherraez@ub.edu (R.H.); 2 Laboratory of Medicinal Chemistry, Faculty of Pharmacy and Food Sciences and Institute of Biomedicine (IBUB), University of Barcelona, 08028 Barcelona, Spain; mpedrola@ub.edu (M.P.); ghashghaei@ub.edu (O.G.); rlavilla@ub.edu (R.L.); 3 Department of Nutrition, Food Science and Gastronomy, Faculty of Pharmacy and Food Sciences, Institute of Biomedicine (IBUB), Institute of Theoretical and Computational Chemistry (IQTC-UB), University of Barcelona, Av. Prat de la Riba 171, 08921 Santa Coloma de Gramenet, Spain; lluis.campos@pharmacelera.com (L.C.-V.); fjluque@ub.edu (F.J.L.); 4 Pharmacelera, Torre R, 4a planta, Despatx A05, Parc Científic de Barcelona, Baldiri Reixac 8, 08028 Barcelona, Spain

**Keywords:** trimethoprim, multidrug-resistant (MDR) bacteria, mechanisms of action, dihydrofolate reductase

## Abstract

This work reports a detailed characterization of the antimicrobial profile of two trimethoprim-like molecules (compounds **1a** and **1b**) identified in previous studies. Both molecules displayed remarkable antimicrobial activity, particularly when combined with sulfamethoxazole. In disk diffusion assays on Petri dishes, compounds **1a** and **1b** showed synergistic effects with colistin. Specifically, in combinations with low concentrations of colistin, very large increases in the activities of compounds **1a** and **1b** were determined, as demonstrated by alterations in the kinetics of bacterial growth despite only slight changes in the fractional inhibitory concentration index. The effect of colistin may be to increase the rate of antibiotic entry while reducing efflux pump activity. Compounds **1a** and **1b** were susceptible to extrusion by efflux pumps, whereas the inhibitor phenylalanine arginyl β-naphthylamide (PAβN) exerted effects similar to those of colistin. The interactions between the target enzyme (dihydrofolate reductase), the coenzyme nicotinamide adenine dinucleotide phosphate (NADPH), and the studied molecules were explored using enzymology tools and computational chemistry. A model based on docking results is reported.

## 1. Introduction

**Figure 1:**
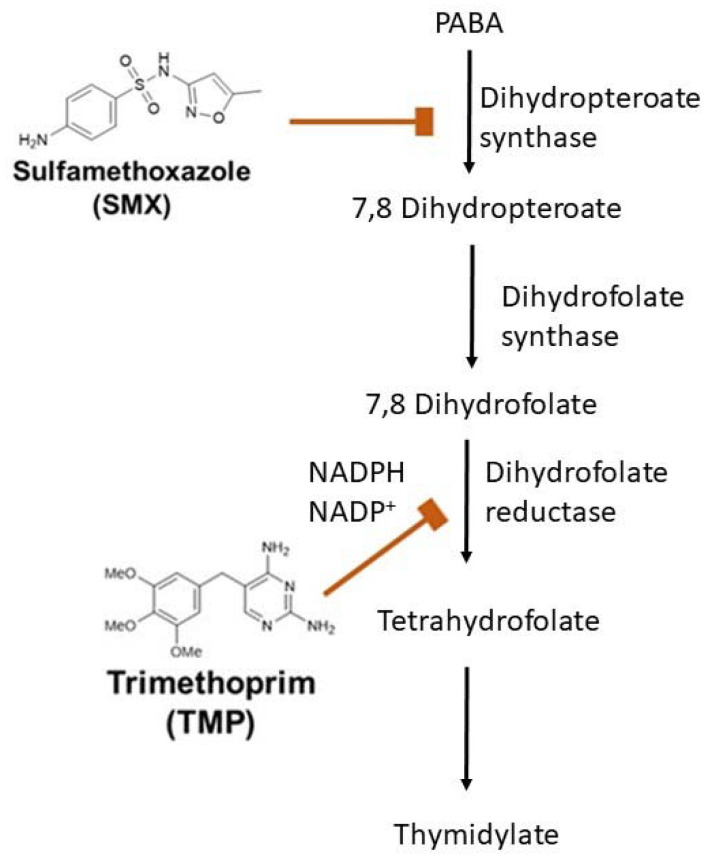
Folic acid biosynthesis pathway.

Unlike eukaryotes, prokaryotes must produce their own nucleotides. In bacteria, a prerequisite for the biosynthesis of thymidine is the production of folate. Trimethoprim (TMP), included in the World Health Organization’s Model List of Essential Medicines, was first used in 1962 and remains a first-line antibiotic in many countries, usually in combination with sulfamethoxazole (SMX), with which it acts synergistically. TMP and SMX target two key enzymes of the folate pathway, acting as inhibitors of dihydrofolate reductase (DHFR) and dihydropteroate synthetase (DHPS), respectively (Figure 1). 

Antimicrobial resistance remains a major challenge for microbiologists and public health officials, as infections by multidrug-resistant bacteria have reached worrisome levels. Resistant bacteria include: (i) multi-resistant strains, which are resistant to antimicrobials from at least three different families, (ii) extremely resistant strains, which are resistant to all antimicrobials except colistin, and (iii) pan-drug-resistant strains, resistant to all available antimicrobials [1].

Among the lines of research aimed at overcoming antimicrobial resistance are: (i) the “green” approach, based on innovative eco-friendly antimicrobials [2], and (ii) the chemical approach, based on the synthesis of new molecules with antimicrobial action, including: (a) inhibitors of the resistance mechanisms to potentiate already existing antimicrobials [3]; (b) bioactive peptides [4], (c) the conjugation of different molecules to generate new ones with improved properties [5], and (d) a combination of two or more of these strategies. The interests of our laboratories include the synthesis of molecules whose active sites resemble those of already known antibiotics [6], the improvement of molecular delivery, such as the use of nanoparticles as antibiotics carriers in nano-medical devices [7], and the modification of natural molecules such that they acquire antimicrobial properties [8].

In this article we examine two TMP-like molecules that have been recently reported [6], focusing on their antibacterial effects and their mechanisms of action. TMP acts directly on dihydrofolate reductase (DHFR, EC 1.5.1.3), which catalyzes the reduction of 7,8-dihydrofolate (H_2_F) to 5,6,7,8-tetrahydrofolate (H_1a_) using NADPH as a cofactor. The conversion requires the transfer of a hydride from C4 of the NADPH cofactor to C6 of the pterin ring of H_2_F, accompanied by the protonation of dihydrofolate on N5. This reaction is essential in the de novo synthesis of purines, thymidine, and certain amino acids [9,10]. Given the important role of DHFR, which is ubiquitously expressed by all kingdoms of life, the enzyme may be an effective therapeutic target in cells with a rapid DNA turnover and therefore both in bacteria and the treatment of cancer [11,12].

**Scheme 1:**
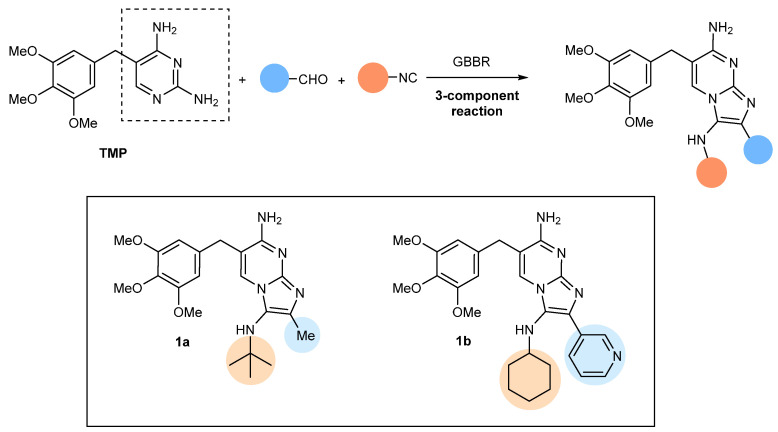
Multicomponent access to new trimethoprim (TMP) derivatives.

Based on the straightforward synthesis of compounds **1a** and **1b**, which were readily prepared through a Groebke-Blackburn-Bienaymé (GBBR) multicomponent reaction (Scheme 1), and the preliminary bacteriological profiles, which combine suitable antibiotic potency and kinetics [6], compounds **1a** and **1b** are explored in this study with respect to their mechanism of action and possible synergistic effects when used in combination with other antibacterial agents.

## 2. Results and Discussion

### 2.1. Antimicrobial Susceptibility of Planktonic Bacteria

The minimum inhibitory concentrations (MICs) of TMP, **1a** and **1b**, both alone and in combination with SMX and colistin, are shown in Table 1. The *E. coli* ATCC 25922 strain was susceptible to TMP, **1a** and **1b**, and the activity was enhanced when the compounds were combined with SMX. The *P. aeruginosa* PAO1 strain was resistant to TMP, **1a** and **1b**, but susceptible to colistin. On the contrary, S. *marcescens* was colistin-resistant, but susceptible to the combined treatment of the compounds with SMX. Finally, **1a** and **1b** were also tested in TMP-resistant strains (*E. coli* 220560529 and *S. epidermidis* 220560752) but no significant effect was observed, suggesting that these compounds should share the target with TMP (data not shown).

### 2.2. Antimicrobial Susceptibility of Sessile Bacteria

Many microorganisms that cause infectious diseases normally grow attached to a surface or an interface, thus forming biofilms. These structured communities contain bacterial cells of one or more species, attached to a living or inert surface and immersed in a hydrated polymeric matrix [13]. Within the biofilm, bacteria grow as part of complex and dynamic systems that result in their ability to tolerate antimicrobials [14], leading to persistent infections. Thus, the efficacy of new antibiotics requires both conventional antimicrobial susceptibility tests against planktonic cells (e.g., MIC determinations) and tests against bacteria residing in biofilms. In this work, the activity of **1a** and **1b** was measured under both conditions and then compared with the corresponding activity of TMP. 

The minimum biofilm eradication concentration (MBEC) and biofilm prevention concentration (BPC) for TMP and compounds **1a** and **1b**, alone and in combination with SMX (1:20), are shown in Table 2. Antimicrobials that exhibited antibiofilm activity had higher MBECs than BPCs. This result reflects the fact that the MBEC is a measure of the antimicrobial activity on mature biofilms, while the BPC is the concentration at which biofilm formation is blocked by the antimicrobial. Compound **1b** was highly active in biofilm prevention, whereas neither TMP nor compounds **1a** and **1b** were able to fully eradicate *Staphylococcus aureus* biofilms (neither *S. aureus* ATCC 29213 nor *S. aureus* 8124825998). 

### 2.3. Synergism Studies

Table 3 shows the results of TMP-SMX checkerboard assays in combination with colistin when tested in four clinical strains: *E. coli* 220560529, *P. aeruginosa* SJD 536, *P. aeruginosa* SJD VH023, and *P. aeruginosa* SJD 481. Synergistic effects between the two drugs were not found, as noted in the values of the fractional inhibitory concentration index (FICi), which is ≥0.5 and <4, in any of the studied strains. 

**Figure 2:**
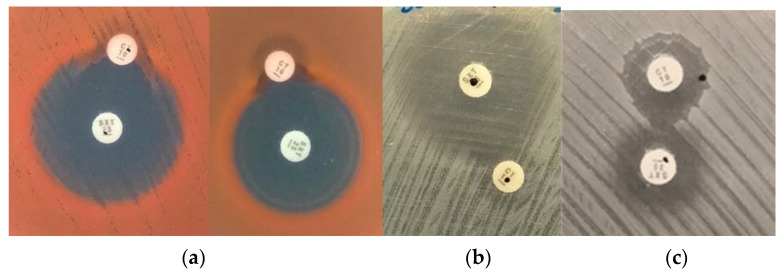
Interaction between colistin and TMP-SMX in (**a**) *S. marcescens* NIMA; (**b**) *E. coli* ATCC 25922, and (**c**) *P. aeruginosa* PAO1.

By contrast, a strong synergism was observed with these drugs in TMP-susceptible/colistin-resistant *S. marcescens* (Table 4). This suggests that the susceptibility to TMP and TMP-like molecules in some gram-negative bacteria is due to limitations in TMP transport through the bacterial outer membrane. Colistin is unable to kill *S. marcescens* but it does adversely impact prodigiosin biosynthesis [15]. Effects on both the entry of antimicrobials as well as drug extrusion by efflux pumps have also been described [16]. Thus, as reported for antimicrobials such as linezolid and rifampin [17], colistin could be used to enhance bacterial susceptibility to TMP and TMP-like compounds. The use of very low concentrations of these drugs would limit their toxicity. It should be noted that in preliminary plate experiments, positive effects between colistin and TMP-SMX were observed in *E. coli* and *P. aeruginosa* (Figure 2), in agreement with the relatively low FICi (close to 1). In colistin-susceptible bacteria, however, the lethality of colistin masked any possible synergy. 

**Figure 3:**
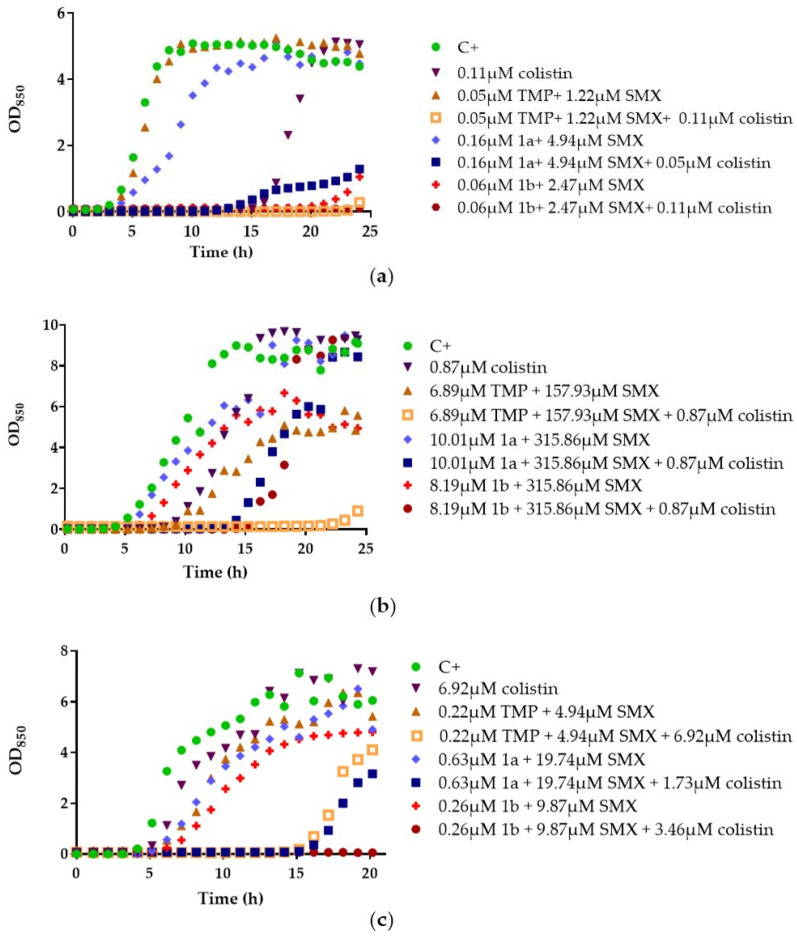
Effects of TMP, **1a** and **1b** when tested in combination with SMX (1:20) and in the presence of sublethal concentrations of colistin on the growth curve of (**a**) *E. coli* ATCC 25922, (**b**) *P. aeruginosa* PAO1, and (**c**) *S. marcescens* NIMA.

The effect of combining TMP, **1a** and **1b** with SMX and colistin in real-time were determined by plotting growth curves for *E. coli* ATCC 25922, *P. aeruginosa* PAO1, and *S. marcescens* NIMA using concentrations at which these strains were fully resistant (Figure 3). When TMP, **1a** and **1b** were used in combination with colistin, bacterial growth was nearly abolished, thus demonstrating synergism between these antibiotics. In the kinetics profile of *E. coli* (Figure 3a), although 0.11 µM colistin provoked a 10 h delay in growth, growth had resumed to the same level as the control at the end of the experiment. Following the addition of TMP + SMX (0.05 µM + 1.22 µM), however, growth was delayed for 20 h.

A similar effect was observed in *P. aeruginosa*, both with TMP and with compounds **1a** and **1b** (Figure 3b). At a colistin dose of 0.87 µM, the growth was delayed for 2 h and then reached the same level as the control. Similar curves were obtained with TMP + SMX (6.89 µM + 157.93 µM), whereas the growth delay was 4 h with **1a** + SMX (10.01 µM + 315.86 µM) and **1b** + SMX (8.19 µM + 315.86 µM). The addition of 0.87 µM colistin to **1a** + SMX and **1b** + SMX resulted in a delay of 10 h, whereas the addition of 0.87 µM colistin to TMP + SMX completely abolished the growth for 23 h after the start of the experiment. The results from the agar plates and the growth curves suggested an additive effect, even though it was not clearly reflected in the FICi values.

The effects of the antibiotics were also explored in *S. marcescens* (Figure 3c), which is intrinsically fully resistant to colistin. Thus, while colistin severely alters the bacterium’s outer membrane, it does not affect bacterial viability, as the cytoplasmic membrane remains intact. The effect of colistin on the outer membrane of Serratia can be readily seen by transmission electron microscopy [18]. The growth curve of *S. marcescens* in the presence of TMP + SMX and colistin exhibited a longer delay (up to 10 h) in the start of detectable growth compared to the delay observed in the presence of TMP + SMX. Similar results were obtained with **1a**. Moreover, when testing **1b**, a complete abolition of growth was obtained in the presence of colistin. 

The nearly complete abolition or prolonged delay of growth in the studied bacteria suggested that colistin alters the hydrophobic permeability barrier of the lipopolysaccharide outer membrane and thus facilitates the internalization of the DHFR inhibitors, which then inhibit the bacterial growth. The demonstration of these synergistic effects in gram-negative bacteria should renew the interest for the use of TMP and TMP-like molecules.

### 2.4. The Role of Efflux Pumps

The TMP analogues were tested in combination with SMX (1:20) in the presence of 20 µg/mL of the efflux pump inhibitor phenylalanine arginyl ß-naphthylamide (PAßN). At this point, it is worth noting that previous studies demonstrated that they have no detectable effect on bacterial growth when used at concentrations up to 40 µg/mL [19]. The MIC values are shown in Table 5. Lower MIC values were obtained in the presence of PAßN compared to the assays performed in the absence of the efflux pump inhibitor. 

A recent study demonstrated that colistin, even at low concentrations, has a direct effect on efflux pump functionality [16]. In the present study, the addition of 20 µg PAßN/mL resulted in a 16-fold reduction in the MICs of TMP and its analogues (Table 5). These results are consistent with the critical role of efflux pumps in bacterial susceptibility to these antimicrobials. 

This work examined possible synergisms between colistin and TMP, **1a** and **1b** in enhancing the antimicrobial activity, following the positive results in preliminary experiments on Petri dishes (Figure 2). The synergistic effects of colistin and all three TMP molecules were confirmed (Figure 3 and Table 4) and a possible mechanism involving bacterial efflux pumps was demonstrated. Our findings suggest that DHFR inhibitors can be used in combination with low concentrations of colistin or similar molecules as a new approach for the treatment of infections caused by multidrug-resistant variants of gram-negative bacteria. The synergism between colistin and the TMP molecules in *S. marcescens* further suggested that peptides with the ability to facilitate antibiotic penetration by altering the bacteria outer membrane and inhibiting bacterial efflux pumps can be used to sensitize bacteria to a wide range of otherwise ineffective antimicrobials.

### 2.5. Enzymatic Assays

**Figure 4:**
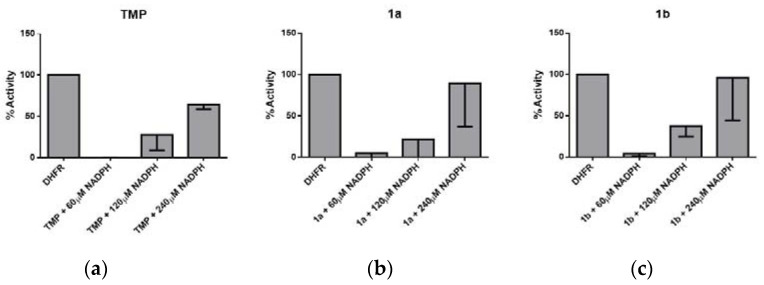
Activity (%) of *E. coli* dihydrofolate reductase (DHFR) after a 30-min incubation with substrate in the presence of the inhibitors (**a**) TMP, (**b**) **1a** and (**c**) **1b**, each at a concentration of 5 µM, and different concentrations of NADPH. The data are expressed as a percentage with respect to the control.

Like TMP, compounds **1a** and **1b** are potent inhibitors of DHFR. Both analogues inhibited the activity of this enzyme by >80%. All three inhibitors were then tested in *E. coli* supplied with increased concentrations of the DHFR cofactor NADPH and the substrate H_2_F. The reaction without inhibitors served as the reference (100% activity). In the reactions with TMP or its analogues, increasing concentrations of NADPH resulted in the increased enzyme activity (Figure 4). The enzyme activity was the highest at the highest tested concentration of NADPH (240 µM).

**Figure 5:**
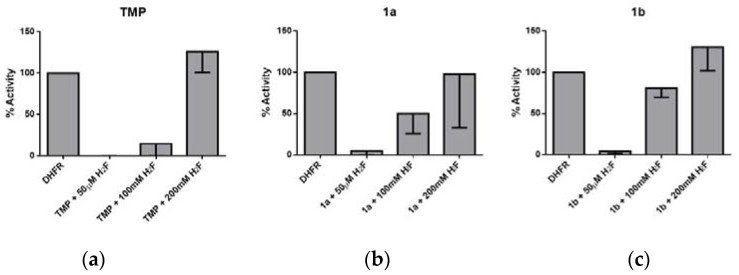
Percentage of activity of *E. coli* DHFR after a 30-min incubation. Each histogram represents the activity of DHFR in the presence of different concentrations of H_2_F and 5 µM of the inhibitors (**a**) TMP, (**b**) **1a** and (**c**) **1b**. The data are expressed as a percentage with respect to the control.

Similar results were obtained for the three antibiotics tested in the presence of increasing concentrations of H_2_F (Figure 5) When the enzymatic assays were performed using a substrate concentration four times higher than the concentration used in the first assay (i.e., 50 μM H_2_F), the inhibition was nearly reversed. Thus, as the concentrations of NADPH or H_2_F were increased, the enzymatic activity was recovered as well, regardless of the presence of the antibiotics.

**Figure 6:**
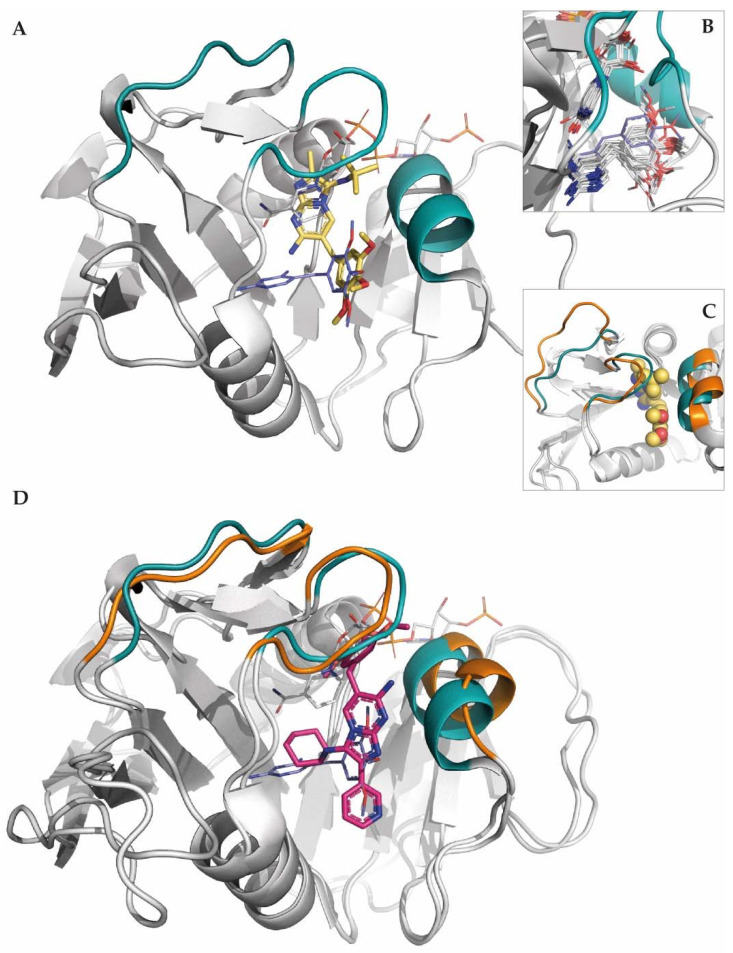
Binding mode of **1a** to the DHFR of *E. coli*. (**A**) The docked positions of **1a** and TMP are shown with their C atoms depicted as yellow sticks and in deep blue, respectively, and with the C atoms of NADPH in gray. (**B**) Superposition of the crystallographic poses of TMP (C atoms in gray). The crystallographic structure of TMP in PDB entry 2W9H, in which the trimethoxybenzene group sterically collides with the nicotinamide ring of NADPH, is shown in deep blue. (**C**) Loops Met20 and F-G, and helix 3 are highlighted to illustrate the differences in the 3D structures of PDB entries 3DAU (turquoise) and 4KM2 (orange). (**D**) The docked position of **1b** (C atoms in magenta) in a homology model of *E. coli* DHFR built using the open structure 4KM2 as a template. The position of TMP and NADPH is shown with their C atoms depicted as deep blue and gray, respectively.

To explore the molecular basis of these results, we examined the binding of **1a** to *E. coli* DHFR. According to the docking model, the heterocyclic ring of **1a** fills the pocket occupied by the nicotinamide ring of NADPH, whereas the trimethoxybenzene ring overlaps the same moiety present in TMP (Figure 6A). Accordingly, the binding of **1a** would compete with the substrate but would also affect the correct alignment of NADPH in its binding pocket. 

This binding model is supported by the following experimental evidence. First, a comparison of the arrangements adopted by TMP in its interaction with human and *E. coli* DHFR showed the similar orientation of the diaminopyrimidine ring, which involves the formation of several hydrogen bonds with residues in the binding pocket (Asp27, Ile5, Ile94). By contrast, the position of the trimethoxybenzene ring is more variable and, in fact, it can adopt multiple arrangements, which often would sterically collide with NADPH when bound to the enzyme. Indeed, the most severe steric hindrance (PDB entry 2W9H; TMP, shown as blue sticks in Figure 6B) would occur in an X-ray structure that did not include NADPH. Furthermore, accommodation of compounds **1a** and **1b** was facilitated by the flexibility of the loops that shape the binding pocket. This was seen in the superposition of the X-ray structures 3DAU (*E. coli*) [20] and 4KM2 (M. tuberculosis) [21], which revealed the altered arrangement of loops Met20 and F-G (Figure 6C), as described in previous studies [22,23]. On the basis of this conformational flexibility, compound **1b** was docked using a structural model of *E. coli* DHFR built using the open structure of the enzyme (PDB entry 4KM2) as a template. The open structure enabled the proper accommodation of **1b** in the binding pocket of *E. coli* DHFR (Figure 6D), which would lead to steric hindrance with the nicotinamide ring of NADPH.

### 2.6. Cytotoxicity

At concentrations as high as 32 μg/mL, which was the maximal concentration considered in these assays, the cytoxicity of TMP, as well as compounds **1a** and **1b** in HepG2 and L-929 cells, was almost negligible (Table 6). A drug is considered toxic when its cytotoxicity level exceeds 20% [24]. For all three compounds, the IC_50_ (the drug concentration needed to inhibit cell growth by 50%) was >32 μg/mL.

## 3. Materials and Methods

### 3.1. Chemical Synthesis

Compounds **1a** and **1b** were prepared as previously reported [6], from the interaction of TMP with aldehydes and isocyanides in acetonitrile under Yb(OTf)_3_ catalysis. The compounds were purified by chromatography and stored at −20 °C under an inert atmosphere. Stock solutions in DMSO were stable when kept in the cold. The integrity of these compounds and solutions thereof was confirmed by HPLC-MS (column: ZORBAX Extend-C18 3.5 μm 2.1 × 50 mm, Agilent; mobile phase A: H_2_O + 0.05% HCOOH; mobile phase B: ACN + 0.05% HCOOH; 10 min; 35 °C). There were no signs of decomposition at least 1 year after their chemical synthesis. 

### 3.2. Antimicrobial Susceptibility of Planktonic Bacteria

The minimum inhibitory concentrations (MICs) of colistin, TMP, **1a**, and **1b** were determined using the microdilution method, according to EUCAST recommendations [25].

### 3.3. Antimicrobial Susceptibility of Sessile Bacteria

TMP and its analogues were tested against biofilms by determining the minimal biofilm eradication concentration (MBEC) and the biofilm prevention concentration (BPC). Bacterial viability within the biofilm was assessed using the dye resazurin, which is reduced by metabolically active bacteria to the fluorescent compound resorufin. Both collection strains, *E. coli* ATCC 25922 and *S. aureus* ATCC 29213, and methicillin-resistant *S. aureus* 8124825998 were tested. All three strains were grown in tryptic soy broth (TSB) with shaking at 200 rpm for 24 h at 37 °C. Bacterial biofilms were formed and treated as follows. Overnight suspensions of each strain were diluted 1/100 in TSB. One hundred µL of each of the adjusted cell suspensions were transferred to the wells of flat-bottomed 96-well microtiter plates (Guangzhou Jet Bio-Filtration Co., Ltd., Mianyang, China) and incubated at 37 °C for 24 h. Eight wells filled only with sterile TSB served as the negative controls. All wells were then gently rinsed with 100 µL of Ringer ¼ solution. The biofilms were exposed to several concentrations of the antimicrobials (leaving 8 wells without antimicrobials as a positive control) and incubated at 37 °C for 24 h. After the wells were again rinsed with 100 µL of Ringer ¼ solution, 100 µL of resazurin (0.0015%) was added to each well. The plates were then incubated for 3–5 h, after which cell fluorescence (λ_ex_ = 530; λ_em_ = 590) was measured using a scanning multi-well spectrophotometer (FLUOstar OPTIMA, BMG Labtech, Ortenberg, Germany)). The MBEC was defined as the lowest concentration of antimicrobial activity that prevented bacterial regrowth from the treated biofilm.

### 3.4. Synergism Studies

The fractional inhibitory concentration (FIC) of TMP-SMX in combination with colistin was determined by the checkerboard method in four clinical bacterial strains: *E. coli* 220560529, *P. aeruginosa* SJD 536, *P. aeruginosa* SJD 481 (all three TMP resistant), and *P. aeruginosa* VH023. In addition, the FICs of colistin with TMP, **1a** and **1b**, and SMX (1:20) were determined in *E. coli* ATCC 25922, *P. aeruginosa* PAO1, and *S. marcescens* NIMA. The assays were performed in 96-well plates using serial dilutions of TMP and SMX (1:20). Serial dilutions of colistin starting from twice the previously determined MIC were prepared and added to plates inoculated with 5 μL of bacterial suspension. The plates were incubated overnight at 37 °C and read after at least 16 h of incubation. 

The fractional inhibitory concentration index (FICi) was determined according to the formula FICi = FIC A + FIC B, where FIC A is the MIC of drug A (TMP + SMX) in combination/MIC of drug A alone and FIC B (colistin) is the MIC of drug B in combination/MIC of drug B alone. The combinations were defined as synergistic (FICi ≤ 0.5), indifferent (FICi > 0.5 and < 4), or antagonistic (FICi ≥ 4). An antimicrobial effect achieved at a drug concentration that was lower when the drug was used in combination with other drugs than alone was considered to be indicative of synergism between the tested antibiotics.

Additionally, the effect of TMP-SMX and the GBBR analogues in combination with sublethal concentrations of colistin was assessed in growth curves of *E. coli* ATCC 25922, *P. aeruginosa* PAO1, and *S. marcescens* NIMA. Exponential-phase cultures were adjusted to 5 × 10^6^ CFU/mL in a final volume of 10 mL of TSB and the antimicrobials were added at sublethal concentrations. Growth was monitored using RTS-1C real-time cell growth loggers (Biosan SIA, Riga, Latvia) in cells incubated for 24 h at 37 °C with shaking at 2000 rpm. Growth was measured every 10 min as the optical density (OD 850 nm).

### 3.5. Efflux Pumps Effect

The efflux pump inhibitor phenyl-arginyl-β-naphthylamide (PaβN) was purchased from Sigma-Aldrich Chemicals (Madrid, Spain). The MIC of the TMP analogues in combination with SMX in the presence of 20 µg PaβN/mL was determined in *P. aeruginosa* PAO1 using the microdilution method. 

### 3.6. Dihydrofolate Reductase Assay

The DHFR assay is based on the reduction of 7,8-dihydrofolate to 5,6,7,8-tetrahydrofolate catalyzed by DHFR and using NADPH as a cofactor. Purified *E. coli* DHFR was kindly provided by E. Shakhnovich and J.V. Rodrigues (Harvard University, Cambridge, MA, USA). The DHFR assay kit (CS0340) was purchased from Sigma-Aldrich. The assay was performed in 96-well flat-bottom plates (Corning Costar 3606, NY, USA) with the protocol adjusted to accommodate a final reaction volume of 200 µL. DHFR was diluted to a final concentration of 0.03 µg/mL. The inhibitory effect of compounds **1a** and **1b** was tested, with TMP serving as the control. All three drugs were used at a concentration of 5 µM, which was higher than the respective MICs (Table 1). To determine the effect of the concentration of the DHFR cofactor NADPH on inhibition, a dilution series of NADPH in assay buffer was carried out to obtain a concentration range between 60 µM and 240 µM. A dilution series of H_2_F was similarly carried out in assay buffer to obtain a concentration range from 50 µM to 200 µM. In all assays, the enzyme was mixed with the different inhibitors and incubated for 30 min before the enzymatic reaction was initiated by the addition of NADPH and H_2_F. The reaction was conducted at 37 °C and monitored by the decrease in absorbance at 340 nm (indicative of a decrease in the NADPH concentration). Measurements were performed every minute for 40 min [26] using a scanning multi-well spectrophotometer (FLUOstar OPTIMA, BMG Labtech, Germany). All measurements were performed in duplicate with three technical replicates.

### 3.7. Computational Chemistry

Docking simulations were carried out to explore the binding mode of TMP, **1a** and **1b** to *E. coli* DHFR, using the 2019–2 release of Glide [27,28]. The crystal structure of *E. coli* DHFR, retrieved from the Protein Data Bank (PDB code 3DAU [20]), includes methotrexate and NADPH. The protein structure of DHFR was thus prepared by deleting both of these compounds, by assigning bond orders, adding hydrogen atoms, and restrained energy minimization, using the Protein Preparation Wizard module in Maestro [29]. Compounds were prepared using LigPrep [30]. The binding site was enclosed in a grid defined with an inner box of 10 Å × 10 Å × 10 Å; GlideScore (SP) was used to evaluate the quality of the configurations [31]. Default settings were used for all remaining parameters. The results of the docking simulations were visually examined with the aid of PyMOL software [32]. Docking of **1b** was performed using a homology model of *E. coli* DHFR using the open structure from *M. tuberculosis* DHFR (PDB entry 4KM2) as a template.

### 3.8. Cytotoxicity

The cytotoxicity assay was carried out in the human hepatocellular carcinoma cell line Hep G2 ATCC and in murine L-929 fibroblasts (NCTC clone 929, ECACC 88102702), based on the experiments described by Vinuesa et al. [23]. The cells were obtained from Dr. Ricardo Pérez-Tomás (Cancer Cell Biology, University of Barcelona). 

The cytotoxicity of TMP and compounds **1a** and **1b** was determined by measuring the intracellular reduction of resazurin sodium salt (Sigma-Aldrich, St. Louis, MO, USA). HepG2 and L-929 cells were grown in RPMI 1640 and MEM medium, respectively (Biochrom AG, Berlin, Germany), supplemented with 10% fetal bovine serum. Cells from pre-confluent cultures were harvested with trypsin-EDTA and maintained at 37 °C and 5% CO_2_. HepG2 and L-929 cells (100 µL each) were seeded in 96-well flat-bottomed microplates to obtain concentrations of 1.5 × 10^4^ and 4 × 10^3^ cells/well, respectively, and incubated at 37 °C for 24 h. Afterwards, the medium was replaced with 200 µL of medium containing the antimicrobials at concentrations ranging from 32 to 0.016 µg/mL and the microplates were incubated at 37 °C for 24 h. Twenty µL of resazurin was then added to each well and the plates were incubated under the same conditions. Fluorescence was measured at an excitation wavelength of 530 nm and an emission wavelength of 590 nm using a scanning multi-well spectrophotometer (FLUOstar OPTIMA, BMG Labtech, Germany). Cytotoxicity was calculated as follows:

(1) $$\%\;\mathrm{Cytotoxicity}\;=\;100-\frac{\left(\mathrm{AT}-\mathrm{ADB}\right)}{\left(\mathrm{AC}-\mathrm{AMB}\right)}\;\times 100$$

where AT is the absorbance of the treated cells, ADB the absorbance of the drug blank control, AC the absorbance of the untreated cells, and ACB the absorbance of the medium blank.

## 4. Conclusions

Two TMP derivatives (**1a** and **1b**) showed antibacterial activity against *E. coli*, *P. aeruginosa* and *S. marcescens* similar to that of TMP and acted synergistically with SMX. They resulted to be active in biofilm prevention, whereas neither TMP nor compounds **1a** and **1b** were able to fully eradicate *S. aureus* biofilms (neither *S. aureus* ATCC 29213 nor *S. aureus* 8124825998). On the other hand, at concentrations at which the products behave as good antibacterials, the cytoxicity on HepG2 and L-929 cell lines was almost negligible. *P. aeruginosa* PAO1 was fully resistant to TMP and its derivatives as well as to the combination of TMP-SMX. Moreover, it can be suggested that blocking their efflux systems may influence the *P. aeruginosa* susceptibility to these antimicrobials. The combination of TMP, TMP-like molecules and SMX with colistin enhances their antimicrobial efficacy against *E. coli, P. aeruginosa* and *S. marcescens* by permeabilizing the cells.

Compounds **1a** and **1b**, like TMP, strongly inhibited the activity of the *E. coli* DHFR. The inhibition was reversed with increasing concentrations of NADPH and H_2_F, suggesting that both molecules interact with the analogues during inhibition. As seen in the docking model, the heterocyclic ring of the compound **1a** fills the pocket occupied by the nicotinamide ring of NADPH. Thus, the binding of **1a** would compete with H_2_F and would also prevent the correct recognition of NADPH.

As the search into new antimicrobial compounds is one of the main pathways to overtake bacterial resistance to antibiotics, it should be emphasized that all putative compounds should be tested in conditions in which the role of the outer membrane as a permeability barrier is inactivated. Their assay together with sublethal concentrations of colistin is proposed as one of the methods of election.

## Figures and Tables

**Table 1:** Minimum inhibitory concentration (MIC, µM) of trimethoprim (TMP) and compounds **1a** and **1b** tested alone and in combination with sulfamethoxazole (SMX) and colistin against *Escherichia coli* ATCC 25922, *Pseudomonas aeruginosa* PAO1, and *Serratia marcescens* NIMA. Data for *P. aeruginosa* PAO1 and *E. coli* ATCC 25922 were already reported [6].

	MIC (µM)		
Antimicrobial	*E. coli* ATCC 25922	*P. aeruginosa* PA01	*S. marcescens* NIMA
TMP	0.43	>110.22	13.78
TMP (SMX)	0.11 (2.37)	13.78 (315.86)	0.86 (19.74)
**1a**	1.25	>80.10	80.10
**1a** (SMX)	0.63 (19.74)	40.05 (1263.43)	2.5 (78.96)
**1b**	1.02	>65.50	16.37
**1b** (SMX)	0.13 (3.95)	32.75 (1263.43)	1.02 (39.48)
Colistin	0.43	1.73	>886.23

**Table 2:** The minimum biofilm eradication concentration (MBEC, μM) and biofilm prevention concentration (BPC, μM) of TMP and the GBBR analogues **1a** and **1b** when tested alone and in combination with SMX (1:20) against *E. coli* ATCC 25922, S. aureus ATCC 29213, and S. aureus 8124825998.

Antimicrobial	*E. coli* ATCC 25922		*S. aureus* ATCC 29213		*S. aureus* 8124825998	
	MBEC (μM)	BPC (μM)	MBEC (μM)	BPC (μM)	MBEC (μM)	BPC (μM)
TMP (SMX)	275.56 (6317.14)	275.56 (6317.14)	>2204.46 (50,537.15)	1102.23 (25,268.58)	>2204.46 (50,537.15)	>2204.46 (50,537.15)
**1a** (SMX)	>1602.06 (50,537.15)	200.26 (6317.14)	>1602.06 (50,537.15)	801.03 (25,268.58)	>1602.06 (50,537.15)	>1602.06 (50,537.15)
**1b** (SMX)	163.74 (6317.14)	81.87 (3158.57)	>1309.91 (50,537.15)	163.74 (6317.14)	>1309.91 (50,537.15)	327.48 (12,634.29)

**Table 3:** Fractional inhibitory concentration index (FICi) of TMP-SMX (1:20) with colistin.

Strains	FICi
*E. coli* 220560529	2.0019
*P. aeruginosa* SJD536	1
*P. aeruginosa* VH023	1
*P. aeruginosa* SJD481	1.003

**Table 4:** FICi of TMP and compounds **1a** and **1b** with SMX (1:20) when tested with colistin.

	FICi		
Antimicrobial	*E. coli* ATCC 25922	*P. aeruginosa* PA01	*S. marcescens* NIMA
TMP + SMX	1.02	1.25	0.13
**1a** + SMX	1.02	1.00	0.25
**1b** + SMX	1.02	1.01	0.25

**Table 5:** MIC values of TMP and compounds **1a** and **1b** in combination with SMX (1:20) as well as colistin in *P. aeruginosa* PAO1 in the presence or absence of the efflux pump inhibitor PAßN (20 µg/mL).

Antimicrobial	MIC (µM)	
	With PAßN	Without PAßN
TMP (SMX)	1.72 (39.48)	27.56 (631.71)
**1a** (SMX)	5.01 (157.93)	80.10 (2526.86)
**1b** (SMX)	2.05 (78.96)	32.76 (1263.43)
Colistin	1.73	1.73

**Table 6:** Cytotoxicity (%) of TMP, **1a** and **1b** in HepG2 and L-929 cells. The data are presented as the percentage of dead cells at maximal concentration tested of the compounds studied (32 µg/mL).

Compound	Cytotoxicity (%)	
	HepG2	L-929
	32 μg/mL	
TMP	0.35	0.8
**1a**	ND	0
**1b**	0.11	0

ND: Not determined.

## Data Availability

Data is contained within the article.
